# Influenza D Virus Infection in Dromedary Camels, Ethiopia

**DOI:** 10.3201/eid2506.181158

**Published:** 2019-06

**Authors:** Shin Murakami, Tomoha Odagiri, Simenew Keskes Melaku, Boldbaatar Bazartseren, Hiroho Ishida, Akiko Takenaka-Uema, Yasushi Muraki, Hiroshi Sentsui, Taisuke Horimoto

**Affiliations:** University of Tokyo, Tokyo, Japan (S. Murakami, T. Odagiri, H. Ishida, A. Takenaka-Uema, T. Horimoto);; Addis Ababa Science and Technology University, Addis Ababa, Ethiopia (S.K. Melaku);; Institute of Veterinary Medicine, Ulaanbaatar, Mongolia (B. Bazartseren);; Iwate Medical University, Iwate, Japan (Y. Muraki);; Nihon University, Kanagawa, Japan (H. Sentsui)

**Keywords:** influenza D virus, viruses, influenza, dromedary, camel, Ethiopia, respiratory infections

## Abstract

Influenza D virus has been found to cause respiratory diseases in livestock. We surveyed healthy dromedary camels in Ethiopia and found a high seroprevalence for this virus, in contrast to animals co-existing with the camels. Our observation implies that dromedary camels may play an important role in the circulation of influenza D virus.

Influenza D virus (IDV) was first isolated from pigs with respiratory symptoms in the United States in 2011 (*1*). Epidemiologic analyses revealed that the most likely main host of IDV is cattle, because the seropositivity rate in these animals is higher than that for other livestock (*2**–**4*). In a recent report, dromedary camels (*Camelus dromedaries*) exhibited substantially high seroprevalence (99%) for IDV in Kenya (*5*), suggesting that this animal is a potential reservoir of IDV. We examined seroprevalence of IDV in dromedary camels in Ethiopia and in Bactrian camels (*Camelus bactrianus*) in Mongolia.

We collected serum samples from dromedary camels (n = 38; average age 4.3 years, range 1–13 years), goats (n = 20; average age 3.9 years, range 1–8 years), sheep (n = 20; average age 2.7 years, range 1–4 years), cattle (n = 15; average age 6.7 years, range, 1–11 years), and donkeys (n = 2; ages 1 and 6) from 2 herds in Bati district, Amhara region, and 1 herd in Fafen district, Somali region, Ethiopia. All animals were apparently healthy, shared the same pasturage during the day, and stayed in barns specific for each animal species at night. To detect influenza D infection, we titrated the serum samples by hemagglutination inhibition (HI) assay using 3 antigenically distinct influenza D strains: D/swine/Oklahoma/1334/2011 (D-OK lineage; D/OK) (*1*), D/bovine/Nebraska/9–5/2013 (D/660-lineage; D/NE) (*6*), and D/bovine/Yamagata/10710/2016 (D/Japan-lineage; D/Yamagata) (*7*). For the HI test, we treated the samples with receptor-destroying enzyme (RDEII; Denka Seiken, http://www.keyscientific.com) at 37°C for 16 h, followed by heat inactivation at 56°C for 30 min. We then reacted serially diluted samples with each virus (4 HAU) at room temperature for 30 min and incubated them with a 0.6% suspension of turkey red blood cells at room temperature for 30 min. The HI titer of each sample was expressed as the reciprocal of the highest sample dilution that completely inhibited HA. We considered samples with HI titer >1:40 positive, to eliminate nonspecific reactions at low dilutions (*4**,**8**,**9*).

Of the 21 dromedary camel samples from Bati, 10 were positive for D/OK, 11 for D/NE, and 19 for D/Yamagata (Figure). Of other animal samples, only 1 goat sample was positive (titer 1:40), indicating that the prevalence rate of influenza D antibodies was higher in dromedary camels than in co-grazing ruminants in the tested herd. The data on the camels’ age indicated that the HI antibodies were not detected due to maternal antibodies, which is only stable for 5–6 months in dromedary camels (*10*). Much closer face-to-face contact may be required for virus transmission among different animal species. The HI titers in camel samples were higher for D/Yamagata (range 1:40–1:160) than those for D/OK and D/NE (1:40–1:80). Meanwhile, we found several positives in dromedary camel samples from Fafen, albeit at lower positive rates and titers compared with those in Bati (Figure). 

**Figure:**
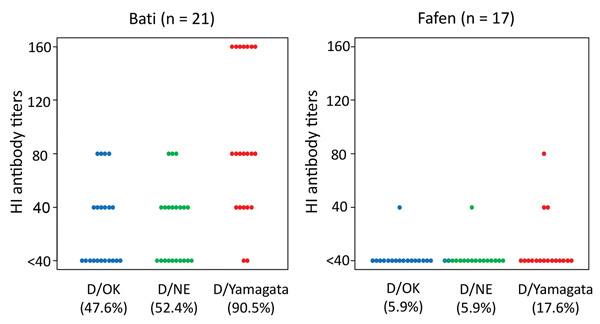
Hemagglutination inhibition (HI) antibody titers for influenza D viruses in serum samples from dromedary camels, Bati and Fafen Districts, Ethiopia. Each dot represents 1 camel. HI assay was performed with RDE(II)-treated serum samples and turkey red blood cells (0.6%) against D/swine/Oklahoma/1334/2011 (D/OK), D/bovine/Nebraska/9–5/2013 (D/NE), and D/bovine/Yamagata/10710/2016 (D/Yamagata). HI-positive rate for each virus is shown below the virus name.

We confirmed the specificity of the HI reaction with a viral neutralizing test using HI-positive samples (data not shown). HI titers obtained were not high, suggesting that the infections may have occurred in these animals some time ago or that results might have been due to the variance of HI methods used in each laboratory. For example, turkey red blood cell was used in this study, whereas horse red blood cell was used in a previous study (*5*). Nonetheless, these data suggest that the virus antigenically related to D/Yamagata was circulating in dromedary camels in this region.

In a previous report, a considerable number of dromedary camels in Kenya were seropositive not only for influenza D but also for influenza C virus (ICV) (*5*). Thus, we additionally used C/Ann Arbor/1/1950 virus as an antigen for HI assay. The results suggest a limited circulation of ICV in this area because 0 samples in Bati were positive and only 2 in Fafen, which were negative for IDV, were positive (titers 1:40). In addition, we performed the HI test using selected samples following preadsorption with ICV. We did not observe any significant decrease in HI titers to IDV, suggesting no cross-reactivity between IDV and ICV in our samples.

We also collected serum samples of apparently healthy Bactrian camels (n = 40) in Dundgovi, Zavkhan, and Umnugovi Provinces, Mongolia, and tested for HI antibody for IDV. These samples did not test positive for these IDV strains.

Despite the limited samples tested, this study suggests that dromedary camels in East Africa might play a substantial role in the circulation of IDV. Further studies using additional samples from multiple countries are expected to clarify the role of this animal on the ecology and epidemiology of this virus, including its reservoir potential in nature.
